# The evolution of assessing bias in Cochrane systematic reviews of interventions: celebrating methodological contributions of the Cochrane Collaboration

**DOI:** 10.1186/2046-4053-2-79

**Published:** 2013-09-23

**Authors:** Lucy Turner, Isabelle Boutron, Asbjørn Hróbjartsson, Douglas G Altman, David Moher

**Affiliations:** 1 Ottawa Hospital Research Institute, Ottawa, Canada; 2 INSERM, U738, Paris, France; 3 Hôpital Hôtel Dieu, Centre, d’Epidémiologie Clinique, Paris, France; 4 Faculté de Médecine, University of Paris Descartes, Sorbonne, Paris Cité, Paris, France; 5 French Cochrane Centre, Paris, France; 6 Nordic Cochrane Centre, Rigshospitalet, Copenhagen, Denmark; 7 Centre for Statistics in Medicine, University of Oxford, Oxford, UK; 8 Department of Epidemiology and Community Medicine, University of Ottawa, Ottawa, Canada

## Background

The global evidence base for health care is extensive, and expanding; with nearly 2 million articles published annually. One estimate suggests 75 trials and 11 systematic reviews are published daily [1]. Research syntheses, in a variety of established and emerging forms, are well recognised as essential tools for summarising evidence with accuracy and reliability [2]. Systematic reviews provide health care practitioners, patients and policy makers with information to help make informed decisions. It is essential that those conducting systematic reviews are cognisant of the potential biases within primary studies and of how such biases could impact review results and subsequent conclusions.

Rigorous and systematic methodological approaches to conducting research synthesis emerged throughout the twentieth century with methods to identify and reduce biases evolving more recently [3,4]. The Cochrane Collaboration has made substantial contributions to the development of how biases are considered in systematic reviews and primary studies. Our objective within this paper is to review some of the landmark methodological contributions by members of the Cochrane Bias Methods Group (BMG) to the body of evidence which guides current bias assessment practices, and to outline the immediate and horizon objectives for future research initiatives.

### Empirical works published prior to the establishment of the Cochrane Collaboration

In 1948, the British Medical Research Council published results of what many consider the first 'modern’ randomised trial [5,6]. Subsequently, the last 65 years has seen continual development of the methods used when conducting primary medical research aiming to reduce inaccuracy in estimates of treatment effects due to potential biases. A large body of literature has accumulated which supports how study characteristics, study reports and publication processes can potentially bias primary study and systematic review results. Much of the methodological research during the first 20 years of The Cochrane Collaboration has built upon that published before the Collaboration was founded. Reporting biases, or more specifically, publication bias and the influence of funding source(s) are not new concepts. Publication bias initially described as the file drawer problem as a bias concept in primary studies was early to emerge and has long been suspected in the social sciences [7]. In 1979 Rosenthal, a psychologist, described the issue in more detail [8] and throughout the 1980s and early 1990s an empirical evidence base began to appear in the medical literature [9-11]. Concurrent with the accumulation of early evidence, methods to detect and mitigate the presence of publication bias also emerged [12-15]. The 1980s also saw initial evidence of the presence of what is now referred to as selective outcome reporting [16] and research investigating the influence of source of funding on study results [10,11,17,18].

The importance of rigorous aspects of trial design (e.g. randomisation, blinding, attrition, treatment compliance) were known in the early 1980s [19] and informed the development by Thomas Chalmers and colleagues of a quality assessment scale to evaluate the design, implementation, and analysis of randomized control trials [20]. The pre-Cochrane era saw the early stages of assessing quality of included studies, with consideration of the most appropriate ways to assess bias. Yet, no standardised means for assessing risk of bias, or “quality” as it was referred to at the time, were implemented when The Cochrane Collaboration was established. The use of scales for assessing quality or risk of bias is currently explicitly discouraged in Cochrane reviews based on more recent evidence [21,22].

### Methodological contributions of the Cochrane Collaboration: 1993 – 2013

In 1996, Moher and colleagues suggested that bias assessment was a new, emerging and important concept and that more evidence was required to identify trial characteristics directly related to bias [23]. Methodological literature pertaining to bias in primary studies published in the last 20 years has contributed to the evolution of bias assessment in Cochrane reviews. How bias is currently assessed has been founded on published studies that provide empirical evidence of the influence of certain study design characteristics on estimates of effect, predominately considering randomised controlled trials.

The publication of Ken Schulz’s work on allocation concealment, sequence generation, and blinding [24,25] the mid-1990s saw a change in the way the Collaboration assessed bias of included studies, and it was recommended that included studies were assessed in relation to how well the generated random sequence was concealed during the trial.

In 2001, the Cochrane Reporting Bias Methods Group now known as the Cochrane Bias Methods Group, was established to investigate how reporting and other biases influence the results of primary studies. The most substantial development in bias assessment practice within the Collaboration was the introduction of the Cochrane Risk of Bias (RoB) Tool in 2008. The tool was developed based on the methodological contributions of meta-epidemiological studies [26,27] and has since been evaluated and updated [28], and integrated into Grading of Recommendations Assessment, Development and Evaluation (GRADE) [29].

**Figure 1:**
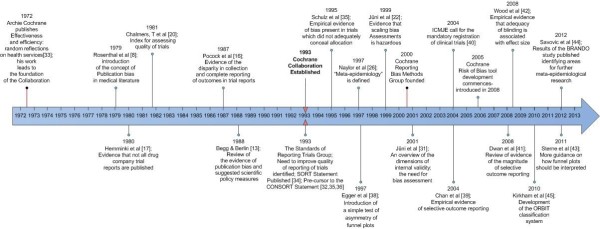
**Timeline of landmark methods research **[8,13,16,17,20,22,26,31-45]**.**

Throughout this paper we define bias as a systematic error or deviation in results or inferences from the truth [30] and should not be confused with “quality”, or how well a trial was conducted. The distinction between internal and external validity is important to review. When we describe bias we are referring to internal validity as opposed to the external validity or generalizability which is subject to demographic or other characteristics [31]. Here, we highlight landmark methodological publications which contribute to understanding how bias influences estimates of effects in Cochrane reviews (Figure 1).

#### Sequence generation and allocation concealment

Early meta-epidemiological studies assessed the impact of inadequate allocation concealment and sequence generation on estimates of effect [24,25]. Evidence suggests that adequate or inadequate allocation concealment modifies estimates of effect in trials [31]. More recently, several other methodological studies have examined whether concealment of allocation is associated with magnitude of effect estimates in controlled clinical trials while avoiding confounding by disease or intervention [42,46].

More recent methodological studies have assessed the importance of proper generation of a random sequence in randomised clinical trials. It is now mandatory, in accordance with the Methodological Expectations for Cochrane Interventions Reviews (MECIR) conduct standards, for all Cochrane systematic reviews to assess potential selection bias (sequence generation and allocation concealment) within included primary studies.

#### Blinding of participants, personnel and outcome assessment

The concept of the placebo effect has been considered since the mid-1950s [47] and the importance of blinding trial interventions to participants has been well known, with the first empirical evidence published in the early 1980s [48]. The body of empirical evidence on the influence on blinding has grown since the mid-1990s, especially in the last decade, with some evidence highlighting that blinding is important for several reasons [49]. Currently, the Cochrane risk of bias tool suggests blinding of participants and personnel, and blinding of outcome assessment be assessed septely. Moreover consideration should be given to the type of outcome (i.e. objective or subjective outcome) when assessing bias, as evidence suggests that subjective outcomes are more prone to bias due to lack of blinding [42,44] As yet there is no empirical evidence of bias due to lack of blinding of participants and study personnel. However, there is evidence for studies described as 'blind’ or 'double-blind’, which usually includes blinding of one or both of these groups of people. In empirical studies, lack of blinding in randomized trials has been shown to be associated with more exaggerated estimated intervention effects [42,46,50].

Different people can be blinded in a clinical trial [51,52]. Study reports often describe blinding in broad terms, such as 'double blind’. This term makes it impossible to know who was blinded [53]. Such terms are also used very inconsistently [52,54,55] and the frequency of explicit reporting of the blinding status of study participants and personnel remains low even in trials published in top journals [56], despite explicit recommendations. Blinding of the outcome assessor is particularly important, both because the mechanism of bias is simple and foreseeable, and because evidence for bias is unusually clear [57]. A review of methods used for blinding highlights the variety of methods used in practice [58]. More research is ongoing within the Collaboration to consider the best way to consider the influence of lack of blinding within primary studies. Similar to selection bias, performance and detection bias are both mandatory components of risk of bias assessment in accordance with the MECIR standards.

#### Reporting biases

Reporting biases have long been identified as potentially influencing the results of systematic reviews. Bias arises when the dissemination of research findings is influenced by the nature and direction of results, there is still debate over explicit criteria for what constitutes a 'reporting bias’. More recently, biases arising from non-process related issues (i.e. source of funding, publication bias) have been referred to as meta-biases [59]. Here we discuss the literature which has emerged in the last twenty years with regards to two well established reporting biases, non-publication of whole studies (often simply called publication bias) and selective outcome reporting.

##### Publication bias

The last two decades have seen a large body of evidence of the presence of publication bias [60-63] and why authors fail to publish [64,65]. Given that it has long been recognized that investigators frequently fail to report their research findings [66], many more recent papers have been geared towards methods of detecting and estimating the effect of publication bias. An array of methods to test for publication bias and additional recommendations are now available [38,43,67-76], many of which have been evaluated [77-80]. Automatic generation of funnel plots have been incorporated when producing a Cochrane review and software (RevMan) and are encouraged for outcomes with more than ten studies [43].A thorough overview of methods is included in Chapter 10 of the Cochrane Handbook for Systematic Reviews of Interventions [81].

##### Selective outcome reporting

While the concept of publication bias has been well established, studies reporting evidence of the existence of selective reporting of outcomes in trial reports have appeared more recently [39,41,82-87]. In addition, some studies have investigated why some outcomes are omitted from published reports [41,88-90] as well as the impact of omission of outcomes on the findings of meta-analyses [91]. More recently, methods for evaluating selective reporting, namely, the ORBIT (Outcome Reporting Bias in Trials) classification system have been developed. One attempt to mitigate selective reporting is to develop field specific core outcome measures [92] the work of COMET (Core Outcome Measures in Effectiveness Trials) initiative [93] is supported by many members within the Cochrane Collaboration. More research is being conducted with regards to selective reporting of outcomes and selective reporting of trial analyses, within this concept there is much overlap with the movement to improve primary study reports, protocol development and trial registration.

#### Evidence on how to conduct risk of bias assessments

Often overlooked are the processes behind how systematic evaluations or assessments are conducted. In addition to empirical evidence of specific sources of bias, other methodological studies have led to changes in the processes used to assess risk of bias. One influential study published in 1999 highlighted the hazards of scoring 'quality’ of clinical trials when conducting meta-analysis and is one of reasons why each bias is assessed septely as 'high’, 'low’ or 'unclear’ risk rather than using a combined score [22,94]. Prior work investigated blinding of readers, data analysts and manuscript writers [51,95]. More recently, work has been completed to assess blinding of authorship and institutions in primary studies when conducting risk of bias assessments, suggesting that there is discordance in results between blind and unblinded RoB assessments. However uncertainty over best practice remains due to time and resources needed to implement blinding [96].

### Complementary contributions

#### Quality of reporting and reporting guidelines

Assessing primary studies for potential biases is a challenge [97]. During the early 1990s, poor reporting in randomized trials and consequent impediments to systematic review conduct, especially when conducting what is now referred to as 'risk of bias assessment’, were observed. In 1996, an international group of epidemiologists, statisticians, clinical trialists, and medical editors, some of whom were involved with establishing the Cochrane Collaboration, published the CONSORT Statement [32], a checklist of items to be addressed in a report of the findings of an RCT. CONSORT has twice been revised and updated [35,36] and over time, the impact of CONSORT has been noted, for example, CONSORT was considered one of the major milestones in health research methods over the last century by the Patient-Centered Outcomes Research Institute (PCORI) [98].

Issues of poor reporting extend far beyond randomized trials, and many groups have developed guidance to aid reporting of other study types. The EQUATOR Network’s library for health research reporting includes more than 200 reporting guidelines [99]. Despite evidence that the quality of reporting has improved over time, systemic issues with the clarity and transparency of reporting remain [100,101]. Such inadequacies in primary study reporting result in systematic review authors’ inability to assess the presence and extent of bias in primary studies and the possible impact on review results, continued improvements in trial reporting are needed to lead to more informed risk of bias assessments in systematic reviews.

#### Trial registration

During the 1980s and 1990s there were several calls to mitigate publication bias and selective reporting via trial registration [102-104]. After some resistance, in 2004, the BMJ and The Lancet reported that they would only publish registered clinical trials [105] with the International Committee of Medical Journal Editors making a statement to the same effect [40]. Despite the substantial impact of trial registration [106] uptake is still not optimal and it is not mandatory for all trials. A recent report indicated that only 22% of trials mandated by the FDA were reporting trial results on clinicaltrials.gov [107]. One study suggested that despite trial registration being strongly encouraged and even mandated in some jurisdictions only 45.5% of a sample of 323 trials were adequately registered [108].

### Looking forward

Currently, there are three major ongoing initiatives which will contribute to how The Collaboration assesses bias. First, there has been some criticism of the Cochrane risk of bias tool [109] concerning its ease of use and reliability [110,111] and the tool is currently being revised. As a result, a working group is established to improve the format of the tool, with version 2.0 due to be released in 2014. Second, issues of study design arise when assessing risk of bias when including non-randomised studies in systematic reviews [112-114]. Even 10 years ago there were 114 published tools for assessing risk of bias in non-randomised studies [115]. An ongoing Cochrane Methods Innovation Fund project will lead to the release a tool for assessing non-randomized studies as well as tools for cluster and cross-over trials [116]. Third, selective reporting in primary studies is systemic [117] yet further investigation and adoption of sophisticated means of assessment remain somewhat unexplored by the Collaboration. A current initiative is ongoing to explore optimal ways to assess selective reporting within trials. Findings of this initiative will be considered in conjunction with the release of revised RoB tool and its extension for non-randomized studies.

### More immediate issues

Given the increase in meta-epidemiological research, an explicit definition of evidence needed to identify study characteristics which may lead to bias (es) needs to be defined. One long debated issue is the influence of funders as a potential source of bias. In one empirical study, more than half of the protocols for industry-initiated trials stated that the sponsor either owns the data or needs to approve the manuscript, or both; none of these constraints were stated in any of the trial publications [118]. It is important that information about vested interests is collected and presented when relevant [119].

There is an on-going debate related to the risk of bias of trials stopping early because of benefit. A systematic review and a meta-epidemiologic study showed that such truncated RCTs were associated with greater effect sizes than RCTs not stopped early, particularly for trials with small sample size [120,121]. These results were widely debated and discussed [122] and recommendations related to this item are being considered.

In addition, recent meta-epidemiological studies of binary and continuous outcomes showed that treatment effect estimates in single-centre RCTs were significantly larger than in multicenter RCTs even after controlling for sample size [123,124]. The Bias in Randomized and Observational Studies (BRANDO) project combining data from all available meta-epidemiologic studies [44] found consistent results for subjective outcomes when comparing results from single centre and multi-centre trials. Several reasons may explain these differences between study results: small study effect, reporting bias, higher risk of bias in single centre studies, or factors related to the selection of the participants, treatment administration and care providers’ expertise. Further studies are needed to explore the role and effect of these different mechanisms.

### Longer term issues

The scope of methodological research and subsequent contributions and evolution in bias assessment over the last 20 years has been substantial. However, there remains much work to be done, particularly in line with innovations in systematic review methodology itself. There is no standardised methodological approach to the conduct of systematic reviews. Subject to a given clinical question, it may be most appropriate to conduct a network meta-analysis, scoping review, a rapid review, or update any of these reviews. Along with the development of these differing types of review, there is the need for bias assessment methods to develop concurrently.

The way in which research synthesis is conducted may change further with technological advances [125]. Globally, there are numerous initiatives to establish integrated administrative databases which may open up new research avenues and methodological questions about assessing bias when primary study results are housed within such databases.

Despite the increase in meta-epidemiological research identifying study characteristics which could contribute to bias in studies, further investigation is needed. For example, as yet there has been little research on integration of risk of bias results into review findings. This is done infrequently and guidance on how to do it could be improved [126]. Concurrently, although some work has been done, little is known about how magnitude and direction in estimates of effect for a given bias and across biases for a particular trial and in turn, set of trials [127].

## Conclusion

To summarise, there has been much research conducted to develop understanding of bias in trials and how these biases could influence the results of systematic reviews. Much of this work has been conducted since the Cochrane Collaboration was established either as a direct initiative of the Collaboration or thanks to the work of many affiliated individuals. There has been clear advancement in mandatory processes for assessing bias in Cochrane reviews. These processes, based on a growing body of empirical evidence have aimed to improve the overall quality of the systematic review literature, however, many areas of bias remain unexplored and as the evidence evolves, the processes used to assess and interpret biases and review results will also need to adapt.

## Competing interests

The authors declare that they have no competing interests.

## Authors’ contributions

This paper was invited for submission by the Cochrane Bias Methods Group. AH and IB conceived of the initial outline of the manuscript, LT collected information and references and drafted the manuscript, DM reviewed the manuscript and provided guidance on structure, DM, DGA, IB and AH all provided feedback on the manuscript and suggestions of additional literature. All authors read and approved the final manuscript.

## Cochrane awards received by BMG members

The Cochrane BMG currently has a membership of over 200 statisticians, clinicians, epidemiologists and researchers interested in issues of bias in systematic reviews. For a full list of BMG members, please login to Archie or visit http://www.bmg.cochrane.org for contact information.

## Bill Silverman prize recipients

**2009** - Moher D, Tetzlaff J, Tricco AC, Sampson M, Altman DG. Epidemiology and reporting characteristics of systematic reviews. PLoS Medicine 2007 4(3): e78. doi: http://dx.doi.org/10.1371/journal.pmed.0040078 [full-text PDF].

## Thomas C. Chalmers award recipients

**2001** (tie) - Henry D, Moxey A, O’Connell D. Agreement between randomised and non-randomised studies - the effects of bias and confounding [abstract]. Proceedings of the Ninth Cochrane Colloquium, 2001.

**2001** (runner-up) - Full publication: Sterne JAC, Jüni P, Schulz KF, Altman DG, Bartlett C, and Egger M. Statistical methods for assessing the influence of study characteristics on treatment effects in “meta-epidemiological” research. Stat Med 2002;21:1513–1524.

**2010** - Kirkham JJ, Riley R, Williamson P. Is multivariate meta-analysis a solution for reducing the impact of outcome reporting bias in systematic reviews? [abstract] Proceedings of the Eighteenth Cochrane Colloquium, 2010.

**2012** - Page MJ, McKenzie JE, Green SE, Forbes A. Types of selective inclusion and reporting bias in randomised trials and systematic reviews of randomised trials [presentation]. Proceedings of the Twentieth Cochrane Colloquium, 2012.
